# Effectiveness of personalized oral health education with behavioural modification using HAPA-MI constructs and oral care kit in residents of informal settlements

**DOI:** 10.1038/s41405-025-00329-5

**Published:** 2025-04-16

**Authors:** Prasad Rath, Rupsa Das, Karishma Rathor, Swagatika Panda

**Affiliations:** 1 Institute of Dental Sciences, Siksha ‘O’ Anusandhan deemed to be University, Bhubaneswar, Odisha India; 2 Department of Oral Medicine and Radiology, Institute of Dental Sciences, Siksha ‘O’ Anusandhan deemed to be University, Bhubaneswar, Odisha India; 3 Department of Public Health Dentistry, Institute of Dental Sciences, Siksha ‘O’ Anusandhan deemed to be University, Bhubaneswar, Odisha India; 4 Department of Oral Pathology and Microbiology, Institute of Dental Sciences, Siksha ‘O’ Anusandhan deemed to be University, Bhubaneswar, Odisha India

**Keywords:** Dentistry, Oral hygiene

## Abstract

**Background:**

Oral health is a crucial determinant of overall well-being, yet ‘residents of informal settlement’, previously referred as ‘slum dwellers’, face significant barriers to maintaining it. Personalized oral health education and behavior modification using models like the Health Action Process Approach (HAPA) and Motivational Interviewing (MI) can address these barriers. While HAPA and MI have shown promise, their individual limitations highlight the need for a combined approach. Integrating these models with personalized education and oral care kits provides a holistic solution to address both motivational and practical barriers. This study evaluates the effectiveness of such an intervention on oral health behaviors and outcomes in residents of informal settlement.

**Methodology:**

A quasi-experimental pre and post interventional study was conducted among 45 participants aged 18–60 years from three wards in Bhubaneswar. The study was conducted between October 24, 2023, to December 24, 2023. Participants were recruited through dental camps organised by our institute. Baseline oral health behavior was assessed using a validated questionnaire based on four behavioral constructs: outcome expectancy (OE), self-efficacy (SE), intention (I), and perceived barriers (PB) by faculty and postgraduate students of public health dentistry department. Participants also received personalized oral health education and an oral care kit. Plaque index (PI), Oral Hygiene Index-Simplified (OHI-S), and behaviour towards oral health were recorded both at baseline (T0) and one-month post-intervention (T1) by same examiners. Statistical analyses included paired t-tests, chi-square tests, and Cronbach’s alpha for internal consistency of the questionnaire, with *p *≤ 0.05 considered significant.

**Results:**

Post-intervention, significant improvements were observed in all behavioral constructs. The mean outcome expectancy (OE) increased from 2.49 ± 0.20 to 4.15 ± 0.07 (*p *= 0.000), self-efficacy (SE) from 1.90 ± 0.14 to 3.81 ± 0.14 (*p *= 0.000), intention (I) from 1.92 ± 0.11 to 4.30 ± 0.33 (*p *= 0.001), and perceived barriers (PB) from 1.85 ± 0.11 to 4.04 ± 0.03 (*p *= 0.002). Clinical outcomes also showed significant improvements: the mean plaque index (PI) decreased from 1.9 ± 0.8 to 0.9 ± 0.4 (*p *= 0.000), and the mean oral hygiene index-simplified (OHI-S) decreased from 2.3 ± 1.4 to 1.5 ± 0.9 (*p *= 0.003). Internal consistency of the questionnaires was good across constructs, with Cronbach’s alpha values ranging from 0.715 to 0.751.

**Conclusion:**

This study demonstrates that a holistic behavioural intervention combining personalized education, behavior modification using HAPA and MI models, and oral care kit distribution significantly improves oral hygiene behavior and clinical outcomes among residents of informal settlement. The model addresses both motivational and access barriers, providing a scalable framework for improving oral health in underserved populations. Future research should explore the long-term sustainability of this approach and its applicability to other settings.

## Introduction

Oral health is essential for overall well-being and quality of life [1]. Regular brushing, flossing, routine dental visits, and a balanced diet are key practices for maintaining oral health. However, inadequate oral hygiene can lead to a variety of oral diseases, such as caries, periodontal disease, and halitosis, which negatively impact physical, social, and psychological well-being. The importance of personalized oral health education has gained recognition as a strategy to promote effective oral hygiene practices. Yet, encouraging sustainable oral hygiene practices is challenging, especially among vulnerable populations, such as residents of informal settlements. These communities face barriers like limited access to oral healthcare services, insufficient awareness of proper oral hygiene, and cultural beliefs that may discourage dental care-seeking behaviors [2]. While oral health education and provision of hygiene kits supply the necessary knowledge and tools, sustainable behavior change requires understanding intentions, perceived barriers, and effective behavior modification. Adopting positive oral health behaviors is central to oral health promotion, as sustainable behavior change supports long-term adherence to healthy habits. Studies show that up to 60% of oral hygiene instructions are forgotten within an hour, and only about half are adhered to when convenient [3, 4]. Various health models have been explored for promoting oral health behaviors, with mixed success [5]. The Health Action Process Approach (HAPA) model is particularly promising, as it addresses the progression from intention to behavior maintenance. HAPA includes two phases: a motivational phase, where individuals consider the risks of poor hygiene, benefits of good oral health, and their own confidence in achieving these goals, followed by a volitional phase, where they plan specific actions and strategies to overcome barriers [6, 7]. Similarly, Motivational Interviewing (MI) is a behavior intervention model that fosters intrinsic motivation for adopting positive habits and maintaining them long-term [8]. Each model, however, has limitations: HAPA emphasizes planning and goal-setting but may overlook motivational and emotional factors, while MI requires skilled practitioners and multiple, time-intensive sessions for effectiveness [9–12]. Recent systematic review has highlighted the importance of combining cognitive behavioral therapies with MI to enhance oral health outcomes [13]. While previous studies have examined the influence of HAPA [14], MI [15], and oral hygiene education [16] separately, there is evidence on their limited effectiveness [7, 8, 17, 18]. There is one study which [19] studied the cumulative reduction in caries incidence among the toddlers by using MI as the main tool to elicit internal motivation which was guided by HAPA theory. Therefore, we hypothesized that combining HAPA with MI along with provision of basic oral care tool can improvise the oral health especially among the underprivileged populations who lack access to oral health awareness programmes and oral health care.

This study aims to assess the effectiveness of a combined intervention comprising personalized oral health education, behavior modification through a custom-designed questionnaire based on HAPA and MI constructs, and the distribution of a basic oral hygiene kit. Through this approach, we intend to improve oral hygiene practices among underprivileged populations, increase compliance with regular brushing and flossing, and reduce plaque and oral hygiene index-simplified (OHI-S) scores. Additionally, we aimed to evaluate the association between behavioral constructs—such as outcome expectancy, self-efficacy, intention, and perceived barriers with these clinical indices, to understand the behavioral shifts that underpin improved oral health practices.

Additionally, this study targets the marginalized urban population living in environments with limited access to resources and basic services. In the past, such groups have been labeled as “slum dwellers”, but this term is now widely recognized as pejorative and stigmatizing. These communities are typically characterized by overcrowded and unplanned settlements, often lacking adequate infrastructure, sanitation, intention and access to formal healthcare services. In this manuscript, we will use the term “residents of informal settlements” to refer to these communities living in precarious conditions. This terminology is considered more respectful and appropriate for describing populations residing in such under-resourced urban areas. Through this combinative approach this study may bridge the gap between intention and sustained behavior change in maintaining oral health in high-risk populations which previous studies have not addressed.

## Methodology

### Study design

This quasi-experimental study assessed the impact of an intervention—comprising personalized oral health education, provision of a basic oral hygiene kit, and behavior modification through a questionnaire based on HAPA and MI constructs—on oral hygiene practices among residents of informal settlement in Bhubaneswar. The study evaluated changes in oral hygiene behaviors and clinical oral health outcomes, such as plaque index and OHI-S, before and after the intervention.

### Type of study

This study primarily involved quantitative data collection and analysis. Pre- and post-intervention data on participants’ behavior and oral health outcomes were collected to determine the intervention’s effectiveness on oral hygiene practices.

### Study site

We conducted oral health screening camps among the residents of informal settlements at three wards of Bhubaneswar, such as ward number 30, 46 and 55. These individuals belong to low-income groups with no or unstable employments such as daily wage labors, street vendors, domestic workers, construction workers etc. Their literacy level is poor. Sanitation and ventilation in those settlements are poor. They have limited access to health care including oral health care, relying mostly on quacks, self-medication or alternative medicine rather than professional dental care. Most importantly nutritional deficiency and lack of autonomy in health relation decisions are prevalent in these settlements.

### Duration of study

This study was conducted over two months, from October 24, 2023, to December 24, 2023, following approval from the Institutional Ethics Committee (Approval No.: EC/NEW/INST/2022/3235).

### Number of subjects

A total of 110 adult participants, aged 18–60, were initially screened. Based on inclusion and exclusion criteria, 45 participants were selected for the study. Post hoc power analysis using G*Power (version 3.1.9.7) revealed a study power of 98% with an effect size of 0.5, a significance level of 0.05, and a sample size of 45 participants. Prior to participation, all eligible individuals were provided with a detailed explanation of the study objectives, procedures, potential benefits, and risks in their native language. Participants were informed that their participation was voluntary and that they could withdraw at any stage without any consequences. Written informed consent was obtained from each participant before enrollment, and for those with limited literacy, verbal consent was recorded in the presence of a witness. To ensure confidentiality, all data collected were anonymized and coded, with no personally identifiable information linked to the research findings. Data were stored securely and were accessible only to the investigators (SP and PR).

#### Inclusion and exclusion criteria

Participants eligible for inclusion in the study were adults aged 18–60 years diagnosed with gingivitis, who were willing to meet all study requirements, possessed the cognitive ability to understand and follow oral hygiene instructions, and had at least 12 teeth, including at least one index tooth to evaluate plaque and OHI-S.

Exclusion criteria included individuals who were current smokers or had quit smoking within the past year, those who were pregnant, or those with systemic diseases that could impact study outcomes, such as diabetes, hypertension, neurological or psychiatric disorders, systemic infections, cancer, or HIV/AIDS. Participants were also excluded if they were on long-term antibiotic therapy, undergoing orthodontic treatment, or diagnosed with periodontitis.

### Sampling method

Convenience or purposive sampling was followed by collaborating with local health clinics, community centers, or NGOs to identify potential residents of informal settlements who gave their consent to participate.

#### Data collection

T0 (baseline): After obtaining informed consent, the baseline oral health status and oral hygiene behavior of all participants (*n *= 45) were assessed by one intern and one faculty member. Data on demographic details (name, age, gender, systemic history, habits), OHI-S [20], and plaque index [21] were recorded. Baseline oral hygiene behavior was assessed using a validated questionnaire based on MI and HAPA constructs. The questionnaire was developed based on established Motivational Interviewing (MI) and Health Action Process Approach (HAPA) frameworks, ensuring theoretical validity. A thorough literature review guided the selection of items relevant to oral hygiene behavior. To establish content validity, a panel of experts in oral health behavior, public health, and behavioral psychology reviewed the questionnaire for clarity, relevance, and completeness, refining it accordingly. It was then pilot-tested on a small sample from the target population to assess comprehensibility and response patterns, with necessary modifications made based on participant feedback. The questionnaire measured four behavioral constructs which included outcome expectancies (OE), self-efficacy (SE), intention (I), and perceived barriers (PB) using items adapted from Schwarzer et al. [6] and Gillam et al. [22]. The constructs consisted of five OE items, six SE items, four I items, and three PB items, each scored on a five-point Likert scale (1 = strongly disagree to 5 = strongly agree). Personalized as well as community based oral health education was provided, and oral hygiene kits were distributed.

#### Interventions

##### Behavior modification intervention

Each selected participant completed a custom-designed questionnaire (Supplementary Table 1) and responses were recorded in the data extraction sheet. The responses to the questionnaires were collected anonymously without linking responses to individual participants. No identifying information was recorded, ensuring complete anonymity in data collection and analysis.

##### Personalized oral health education

Education was delivered at both community and individual levels. Community-level workshops included motivational talks on oral hygiene’s benefits, proper brushing techniques, oral health’s role in systemic health, lifestyle modifications, and dietary influences on oral health The lectures were delivered by faculties and post graduate dental students of community dentistry department outside those settlements addressing small groups of 15 people in three sessions. Audiovisual aids through power point presentation were used to demonstrate the brushing and flossing techniques. At the individual level, one-on-one sessions were tailored to each participant’s specific needs, covering brushing, flossing, and mouthwash use. One on one session was also taken by the faculties and post graduate dental students of community dentistry department.

##### Distribution of oral care kit

Each participant received an oral hygiene kit, including a toothbrush, toothpaste, and dental floss.

T1 (Follow up results): After one month, participants were re-evaluated to assess changes in plaque index, OHI-S, and behavioral constructs using the same custom-designed questionnaire from the baseline.

**Fig. 1:**
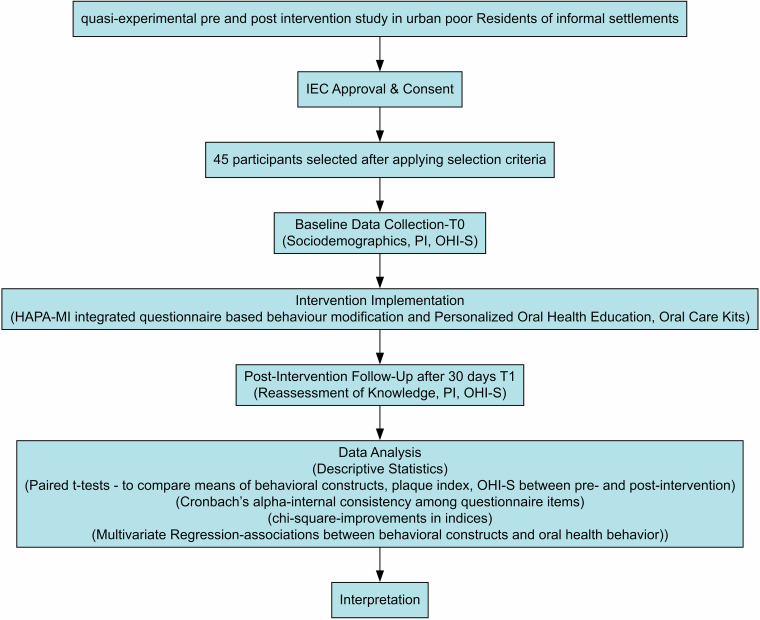
Schematic description of the methodology.

We have depicted the methodology schematically in Fig. 1.

#### Statistical analysis

Data analysis was conducted using SPSS for Mac (version 28, IBM Corporation, Armonk, NY, USA). A *p*-value ≤ 0.05 was considered statistically significant. Categorical variables, such as age, gender, and habits, are presented as frequencies and percentages. Continuous variables, including plaque index, OHI-S, and questionnaire items, are presented as means. The baseline oral health behavior of residents of informal settlements was assessed by summarizing Likert scale responses for each item in the constructs. Paired t-tests compared the means of behavioral constructs, plaque index, and OHI-S between pre- and post-intervention groups. Cronbach’s alpha measured the internal consistency among questionnaire items, while chi-square tests examined improvements in index grades and associations between behavioral constructs and oral health behavior at T0 and T1. Post-intervention, each behavioral construct was assessed in three categories. If the post-intervention score is more than pre-intervention score, then improvement exists. If both scores are equal or post intervention score is less than pre-intervention score, then no improvement exist. The differences in post and pre-intervention score of PI and OHI-S were calculated. To evaluate the influence of change in behavioral constructs on differences in plaque index and OHI-S we conducted multivariate regression analysis.

## Results

### Internal consistency assessment

The internal consistency of the questionnaire items was assessed using Cronbach’s alpha, with scores indicating good reliability across all constructs: outcome expectancy (α = 0.751), self-efficacy (α = 0.715), intention (α = 0.740), and perceived barriers (α = 0.747). These values demonstrated a satisfactory level of internal consistency, supporting the reliability of the measures used in the study.

### Descriptive analysis

The study comprised of 45 participants who gave consent to participate. The mean age among the study participants was 40.13 ± 11.91. The socio-demographic details are described in Table 1. Majority (*n *= 17) of the subjects belonged to the age group of 31–45 years of age followed by participants aged 46–60 years. Males comprised of 51.1% of the study population. The habit of tobacco consumption was seen among 66.7% of participants.

**Table 1:** Socio-demographic details.

Variables	Frequency	Percentage
Age		
18–30	12	26.7
31–45	17	37.8
46–60	16	35.5
Gender		
Female	22	48.9
Male	23	51.1
Tobacco chewing habits		
No habit	15	33.3
yes	30	66.7

### Baseline oral hygiene behavior

Baseline responses across the behavioral constructs revealed low outcome expectancy and self-efficacy in practicing good oral hygiene. Only 4.4–13.3% of participants strongly agreed with statements about the positive effects of brushing, flossing, and the harmful impact of tobacco, while 2.2–6.7% agreed with these statements. Regarding self-efficacy, 0–11.1% strongly agreed on following proper brushing and flossing techniques, while 33.3–48.9% were neutral. Additionally, most participants displayed low intentions for regular oral hygiene practices, with 42.2% disagreeing that improved oral hygiene could benefit systemic health.

### Behavioral constructs post-intervention

The intervention resulted in significant improvements across all items in the four behavioral constructs. Mean scores increased post-intervention for outcome expectancy, self-efficacy, intention, and perceived barriers, with statistical significance (*p *= 0.000) observed for all items. Pre- and post-intervention mean scores are detailed in Table 2.

**Table 2:** Pre- and post-intervention mean scores of behavioral constructs show *p*-values as <0.001.

	Pre-intervention		Post-intervention		*p* value
Outcome expectancy (OE)	Mean	S.D	Mean	S.D	
If I brush my teeth regularly I expect to be free from tooth and gum diseases (OE1)	2.4222	0.89160	4.0444	0.76739	<0.001
If I floss my teeth regularly I expect to be free from gingivitis and periodontitis (OE2)	2.2000	1.07872	4.2222	0.73512	<0.001
If I do not brush my teeth regularly, I perceive the risk of tooth and gum diseases (OE3)	2.6222	0.96032	4.2000	0.72614	<0.001
If I do not floss my teeth regularly I perceive the risk of gingivitis and periodontitis (OE4)	2.5111	1.16037	4.1333	0.75679	<0.001
Tobacco habits can affect teeth and gums adversely (OE5)	2.7333	1.15601	4.1778	0.74739	<0.001
Self - efficacy (SE)					
Are you confident that you are following right brushing technique? (SE1)	1.7778	0.99747	3.7778	0.84984	<0.001
Are you confident that you are following right flossing technique? (SE2)	2.0222	1.21522	3.9333	1.23215	<0.001
Are you willing to give up your habits for better oral health? (SE3)	1.8000	0.66058	3.5778	1.43794	<0.001
Brushing teeth twice daily is good oral hygiene practice (SE4)	2.0667	1.25045	3.7556	0.82999	<0.001
Flossing teeth daily is good oral hygiene practice (SE5)	1.7556	0.67942	3.8444	0.87790	<0.001
We should visit dental hospital/clinic once in every 6 months (SE6)	2.0222	0.94120	4.0000	0.70711	<0.001
Intention (I)					
Do you intend to improve your oral hygiene habits that will improve not only oral health but also systemic health (I1)	2.0222	0.81153	4.4000	0.53936	<0.001
Do you intend to brush twice a day and floss once a day which will prevent oral diseases (I2)	1.8444	0.67270	4.0000	0.95346	<0.001
Do you intend to believe that you need to visit to dentist every 6 months for regular check up (I3)	2.0222	0.75344	4.0889	0.82082	<0.001
Do you know intend to visit the dentist in addition to toothache when you have the complaint of gum bleeding, ulcer or bad breath etc. (I4)	1.8000	0.78625	4.7333	0.44721	<0.001
Perceived barriers (PB)					
Do you know that you need to brush immediately after having sticky food? (PB1)	1.7333	0.80904	4.0889	0.76343	<0.001
Are you aware that eating fiber-rich food keep you protected from dental disease? (PB2)	1.8889	0.88478	4.0222	0.72265	<0.001
In addition to toothbrush we need to use dental aids specific to our requirements like floss, mouthrinse, interdental brushes etc (PB3)	1.9556	0.99899	4.0226	0.72265	<0.001

**Fig. 2:**
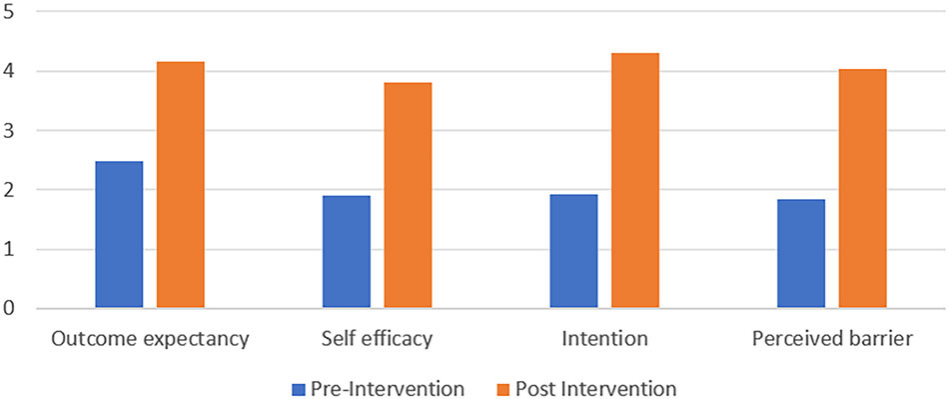
Post-intervention improvement of behavioral constructs.

The total mean outcome expectancy score rose from 2.49 ± 0.20 at baseline to 4.15 ± 0.07 post-intervention. Self-efficacy increased from 1.90 ± 0.14 to 3.81 ± 0.14, intention from 1.92 ± 0.11 to 4.30 ± 0.33, and perceived barriers from 1.85 ± 0.11 to 4.04 ± 0.03, all with highly significant *p*-values (*p *< 0.05) (Table 3, Fig. 2).

**Table 3:** Paired T test of the behavioral constructs.

Behavior constructs	Pre-intervention		Post-intervention		*p* value
	Mean	S.D	Mean	S.D	
Outcome expectancy (OE)	2.49	0.20	4.15	0.07	<0.001*
Self efficacy (SE)	1.90	0.14	3.81	0.14	<0.001*
Intention (I)	1.92	0.11	4.30	0.33	<0.001*
Perceived barrier (PB)	1.85	0.11	4.04	0.03	0.002*

*P < 0.001 - Very significant.

### Post-intervention change in clinical indices

**Fig. 3:**
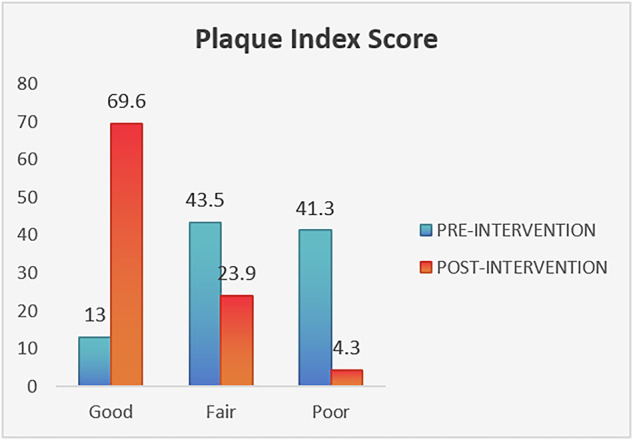
Change in plaque index.

**Fig. 4:**
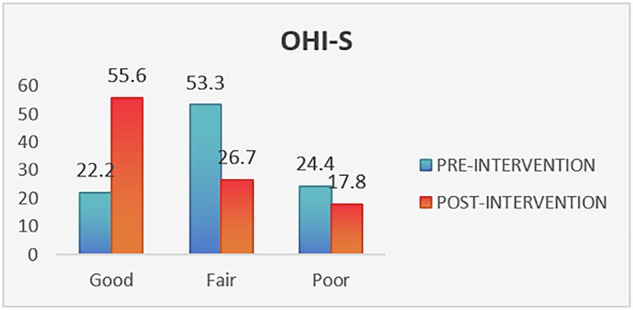
Change in OHI-S index.

The intervention yielded significant improvements in clinical indices. The mean plaque index decreased from 1.9 ± 0.8 at baseline to 0.9 ± 0.4 post-intervention, and the mean OHI-S score declined from 2.3 ± 1.4 to 1.5 ± 0.9. Both reductions were statistically significant (*p *= 0.000 for plaque index and *p *= 0.003 for OHI-S), as presented in Table 4 and Figs. 3 and 4.

**Table 4:** Change in PI and OHI-S after intervention.

	Pre-intervention		Post-intervention		Paired differences^a^		
	Mean	S.D	Mean	S.D	Mean	S.D	*p* value
Plaque index	1.9	0.8	0.9	0.4	0.1	0.938	0.000
Oral hygiene index	2.3	1.4	1.5	0.9	0.4	1.455	0.003

^a^Paired T test.

### Grade-wise improvement in indices

While the plaque index and OHI-S scores showed overall improvement, the change in grade (categorized as good, fair, and poor) was not statistically significant (Chi square *P* value = 0.328 for PI and 0.642 for OHI-S) across all groups.

### Association between behavioral constructs and clinical indices

Pre- and post-intervention analysis revealed positive associations between all behavioral constructs and the clinical indices (plaque index and OHI-S), though these associations did not achieve statistical significance in most items, with the exception of the second item of intention (I2), which was significantly associated with plaque index post-intervention (*p *= 0.03). Supplementary Tables 1 and 2 summarized these associations.

### Multivariate regression analysis to evaluate the effect of change in behavioral constructs on change in PI and OHI-S

The mean difference in PI and OHI-s were found to be 0.13 ± 0.94 and 0.36 ± 1.45 respectively. These values suggested a slight improvement in both the indices. High standard deviations suggested a large variability. Overall model significance in this test did not show any significant effect (Pillai’s Trace value = 0.201, *p *= 0.82; Wilks’ Lambda value = 0.81, *p *= 0.83) of behavioral constructs on either PI or OHI-S t. Individual predictor significance in this test showed that SE1 has a potential effect on OHI-S, though not significant (*p *= 0.09). SE5 (F value = 2.53), PB3 (F value = 2.86) and I4 (F value = 2.49) have moderate effect size, though none were statistically significant. Rest of the behavioral constructs did not affect any of the indices. R² values showed that behavioral constructs explain some variance (28.4% for PI, 31.7% for OHI-S), but the models have weak predictive power.

## Discussion

This study evaluated the effectiveness of a comprehensive intervention combining personalized oral hygiene education, behavior modification using HAPA and MI constructs, and provision of a basic oral care kit among residents of informal settlements of Bhubaneswar, the capital city of Odisha. Our results indicate significant improvements in oral hygiene behavior which was reflected in improvement of plaque index and OHI-S, supporting the hypothesis that a multifaceted approach can effectively enhance oral health practices among the under-privileged population in low-resource settings.

In line with our hypotheses, the study indicated that merging behavior modification strategies with direct oral health education and access to basic oral care tools yields a sustainable impact. Key behaviors, such as brushing frequency, flossing, intention to quit tobacco, dietary choices, and intention to maintain regular dental visits, showed positive shifts across the analyzed constructs. These findings support the idea that addressing motivational and practical barriers holistically can enhance long-term oral hygiene adherence.

Almabadi et al. [23] assessed the joint effectiveness of personalized oral health education and routine dental care and found limited success in this combined approach. They attributed this to the low socioeconomic status of their participants, which may have hindered behavior change during the interventions. To address this challenge, we included the distribution of basic oral hygiene kits to facilitate observable improvements in clinical outcomes. These visible changes could then motivate residents of informal settlement to adopt and sustain positive oral health behaviors. The observed Cronbach’s alpha range for OE, SE, I, and PB were 0.751, 0.715, 0.740 and 0.747 respectively which indicated that the reliability of the measurement instruments used in this study is good and there is an appreciable level of internal consistency across all constructs.

The study population, characterized by low socioeconomic status, limited education, and insufficient knowledge on oral hygiene, highlights the need for targeted interventions among underprivileged populations. These individuals are often at higher risk for oral health issues and less likely to adopt preventive practices. The approach used in this study could be adapted for similar at-risk groups, focusing on both individual and community-level teaching and kit distribution to maximize outreach and impact.

The pragmatic approach of the present study may enable adaptation of this intervention model in underprivileged population who have limited knowledge and access to oral health care. Unlike the other oral health care education studies conducted in low socioeconomic populations [23, 24], the attrition rate in the present study is only 6.25% which further supports the generalizability of this intervention.

The significant improvement in all items of SE construct indicated a boost in the participants’ confidence to perform oral hygiene practices which may lead to sustained behavior change. Similarly, the significant improvement in OE construct explained the strong belief in benefits of appropriate oral hygiene practices which would motivate them to maintain the practice further. Observing the improvement in oral health behavior in the construct, PB, reflected the effectiveness of this mixed intervention in overcoming barriers to oral hygiene practices. The change in responses in the construct, I, highlighted the impact of this intervention on motivation and plans for adapting good oral hygiene practices.

There is also a significant improvement of plaque and OHI-S indices when compared between the pre and post intervention data. Similar results are previously obtained by Neves et al. [25] and Saffari et al. [26] who evaluated the efficacy of MI in reducing plaque and bleeding indices among adult users of health strategies and in reducing scores of gingival inflammation index in pregnant women respectively. However, on studying the association of the behavioral constructs upon the PI and OHI-S did not show any significant impact except the intention construct, although there exists a positive linear association between the two. The statistical significance may be the function of the small sample size and small follow up interval. The multivariate regression analysis suggested the changes in behavioral constructs SE1 had an effect on OHI-S, though it did not reach statistical significance. This highlights the potential role of self-efficacy in influencing oral hygiene practices, particularly in relation to brushing technique awareness. The effect of change in rest of the behavioral constructs weakly influenced the clinical indices. Future exploration with increased sample size, additional covariates, and refined behavioral measures may improve the predictive accuracy. The positive effect of self-efficacy on oral health has also been reported by three previous studies [27–29].

The present study’s strength resides in its amalgamation of three interventions, each independently validated for their efficacy in fostering and sustaining oral health behavior. However, several limitations warrant consideration. Firstly, follow-up data were collected only after one month, potentially limiting the demonstration of significant improvements in clinical outcomes and behavioral changes. Secondly, the sole follow-up session post-intervention allowed for limited interaction time between participants and dentists, possibly impeding optimal outcomes. Therefore, we may suggest that future research should incorporate longitudinal assessments at 3, 6, and 12 months to evaluate behavior retention and relapse rates. Additionally, reinforcing interventions, such as periodic follow-up sessions or digital reminders, could be explored to sustain oral health behavior. Thirdly, the absence of a control group precludes comparative assessments of outcomes. However, to mitigate this limitation, we implemented a quasi-experimental pre-post study design, where each participant served as their own control. Baseline (T0) and post-intervention (T1) assessments allowed us to evaluate changes in behavioral constructs and clinical indices. The statistical significance of these changes strongly suggests the effectiveness of the intervention. Future research incorporating a randomized controlled design with a control group would further validate the intervention’s effectiveness. Although the post hoc power analysis indicated a study power of 98% with an effect size of 0.5 and a significance level of 0.05, the sample size of 45 participants may limit the generalizability of our findings. Future research should aim to recruit a larger and diverse samples to enhance the external validity of the study. Association of these behavioral constructs with improvements in additional clinical parameters such as bleeding on probing, and gingival inflammation can be looked for in future studies which may require long follow up periods.

There are several confounding factors which may have influenced the present result. Low income, low education, cultural beliefs, dietary practices, and lack of access to healthcare are some of the possible factors. For instance, low educational level among the participants may have influenced their initial understanding of oral hygiene practices, affecting the degree of change observed even after the intervention. Additionally, cultural norms and traditional beliefs including use of home remedies or reliance on quacks for oral health care, may have affected compliance with the recommended hygiene practices. Financial constraints and competing priorities like daily livelihood concerns could also limit participants’ ability to sustain improved oral hygiene behaviors beyond the intervention period.

Future studies should incorporate qualitative assessments to better understand the role of these external factors in behavior modification and intervention effectiveness.

## Conclusion

In conclusion, this study demonstrates that a holistic approach, integrating behavior modification, personalized education, and resource provision, effectively improves oral hygiene behaviors and clinical health outcomes among underserved populations. Future studies could explore the long-term sustainability of this model, ideally employing larger sample sizes to further validate its applicability and scalability. To the best of our knowledge, this study is first of its kind, which has the potential to mitigate oral health inequalities and enhance overall well-being in underprivileged populations, aligning with broader public health agendas.

## Supplementary information

Supplemental information

## Data Availability

Available - requests for materials should be addressed to swagatikapanda@soa.ac.in
