# Supplementary material for: Effectiveness of personalized oral health education with behavioural modification using HAPA-MI constructs and oral care kit in residents of informal settlements
Source: BDJ Open. 2025 Apr 16;11:38. doi: 10.1038/s41405-025-00329-5 (PMC12000449; doi:10.1038/s41405-025-00329-5)
Supplement: Supplementary file 1 — Supplemental information [file 41405_2025_329_MOESM1_ESM.docx]

**Demographics and Oral Examination**

Name age/gender

Contact no

Habit

Systemic history – HTN/Diabetes clinical diagnosis -

**Plaque Index**

| **Tooth number** | **mesiofacial** | **distofacial** | **Buccal** | **lingual** |
| --- | --- | --- | --- | --- |
| **12** |  |  |  |  |
| **16** |  |  |  |  |
| **24** |  |  |  |  |
| **32** |  |  |  |  |
| **36** |  |  |  |  |
| **44** |  |  |  |  |

Scoring criteria

0 – no plaque

1 – a film of plaque adhereing to free gingival margin and adjacent area of tooth cannot be seen with naked eye, but by either disclosing solution or probe

2 – moderate accumulation of plaque within the gingival pocket, on the gingival margin or adjacent tooth, can be seen with naked eye

3 – abundance of soft matter within gingival pocketand/or on the tooth and gingival margin

**Simplified Oral hygiene index**

| **Tooth number** |  | **D** | **C** | **D+C** |
| --- | --- | --- | --- | --- |
| **16** | **Facial** |  |  |  |
| **11** | **Facial** |  |  |  |
| **26** | **Facial** |  |  |  |
| **31** | **Facial** |  |  |  |
| **36** | **Lingual** |  |  |  |
| **46** | **Lingual** |  |  |  |

Scoring criteria

Debris (D) Score:

0 - No debris or stain visible on the tooth surface.

1 - Debris or stain covering not more than one-third of the tooth surface.

2 - Debris or stain covering more than one-third but not more than two-thirds of the tooth surface.

3 - Debris or stain covering more than two-thirds of the tooth surface.

Calculus (C) Score:

0 - No calculus or stain visible on the tooth surface.

1 - Supragingival calculus or stain covering not more than one-third of the tooth surface.

2 - Supragingival calculus or stain covering more than one-third but not more than two-thirds of the tooth surface.

3 - Supragingival calculus or stain covering more than two-thirds of the tooth surface.

|  |  | **PRE-INTERVENTION** | | | |  | **POST -INTERVENTION** | | | |  |
| --- | --- | --- | --- | --- | --- | --- | --- | --- | --- | --- | --- |
|  |  | **PLAQUE INDEX** | | | |  | **PLAQUE INDEX** | | | |  |
| **Behavioural constructs** | Likert  Scale score | **Good**  **n(%)** | **Fair**  **n(%)** | **Poor**  **n(%)** | **Total**  **n(%)** | **P value** | **Good**  **n(%)** | **Fair**  **n(%)** | **Poor**  **n(%)** | **Total**  **n(%)** | **P value** |
| OE1 | 1 | 0( 0 ) | 2( 10 ) | 3( 15.8 ) | 5( 11.1 ) | .714 |  |  |  |  | .132 |
|  | 2 | 3( 50 ) | 9( 45 ) | 9( 47.4 ) | 21( 46.7 ) |  | 0( 0 ) | 1( 9.1 ) | 0( 0 ) | 1( 2.2 ) |  |
|  | 3 | 3( 50 ) | 7( 35 ) | 6( 31.6 ) | 16( 35.6 ) |  | 8( 25 ) | 0( 0 ) | 1( 50 ) | 9( 20 ) |  |
|  | 4 | 0( 0 ) | 0( 0 ) | 1( 5.3 ) | 1( 2.2 ) |  | 14( 43.8 ) | 8( 72.7 ) | 0( 0 ) | 22( 48.9 ) |  |
|  | 5 | 0( 0 ) | 2( 10 ) | 0( 0 ) | 2( 4.4 ) |  | 10( 31.3 ) | 2( 18.2 ) | 1( 50 ) | 13( 28.9 ) |  |
| OE2 | 1 | 2( 33.3 ) | 4( 20 ) | 8( 42.1 ) | 14( 31.1 ) | .750 | - | - | - | - | .233 |
|  | 2 | 2( 33.3 ) | 6( 30 ) | 6( 31.6 ) | 14( 31.1 ) |  | - | - | - | - |  |
|  | 3 | 2( 33.3 ) | 7( 35 ) | 4( 21.1 ) | 13( 28.9 ) |  | 7( 21.9 ) | 1( 9.1 ) | 0( 0 ) | 8( 17.8 ) |  |
|  | 4 | 0( 0 ) | 1( 5 ) | 1( 5.3 ) | 2( 4.4 ) |  | 12( 37.5 ) | 7( 63.6 ) | 0( 0 ) | 19( 42.2 ) |  |
|  | 5 | 0( 0 ) | 2( 10 ) | 0( 0 ) | 2( 4.4 ) |  | 13( 40.6 ) | 3( 27.3 ) | 2( 100 ) | 18( 40 ) |  |
| OE3 | 1 | 0( 0 ) | 1( 5 ) | 4( 21.1 ) | 5( 11.1 ) | .517 | - | - | - | - | .402 |
|  | 2 | 2( 33.3 ) | 7( 35 ) | 6( 31.6 ) | 15( 33.3 ) |  | - | - | - | - |  |
|  | 3 | 4( 66.7 ) | 8( 40 ) | 7( 36.8 ) | 19( 42.2 ) |  | 6( 18.8 ) | 1( 9.1 ) | 1( 50 ) | 8( 17.8 ) |  |
|  | 4 | 0( 0 ) | 2( 10 ) | 2( 10.5 ) | 4( 8.9 ) |  | 13( 40.6 ) | 7( 63.6 ) | 0( 0 ) | 20( 44.4 ) |  |
|  | 5 | 0( 0 ) | 2( 10 ) | 0( 0 ) | 2( 4.4 ) |  | 13( 40.6 ) | 3( 27.3 ) | 1( 50 ) | 17( 37.8 ) |  |
| OE 4 | 1 | 1( 16.7 ) | 3( 15 ) | 5( 26.3 ) | 9( 20 ) | .730 | - | - | - | - | **.038** |
|  | 2 | 2( 33.3 ) | 7( 35 ) | 6( 31.6 ) | 15( 33.3 ) |  | - | - | - | - |  |
|  | 3 | 2( 33.3 ) | 7( 35 ) | 5( 26.3 ) | 14( 31.1 ) |  | 6( 18.8 ) | 2( 18.2 ) | 2( 100 ) | 10( 22.2 ) |  |
|  | 4 | 1( 16.7 ) | 0( 0 ) | 2( 10.5 ) | 3( 6.7 ) |  | 12( 37.5 ) | 7( 63.6 ) | 0( 0 ) | 19( 42.2 ) |  |
|  | 5 | 0( 0 ) | 3( 15 ) | 1( 5.3 ) | 4( 8.9 ) |  | 14( 43.8 ) | 2( 18.2 ) | 0( 0 ) | 16( 35.6 ) |  |
| OE 5 | 1 | 0( 0 ) | 1( 5 ) | 4( 21.1 ) | 5( 11.1 ) | .811 | - | - | - | - | .322 |
|  | 2 | 2( 33.3 ) | 7( 35 ) | 7( 36.8 ) | 16( 35.6 ) |  | - | - | - | - |  |
|  | 3 | 3( 50 ) | 8( 40 ) | 5( 26.3 ) | 16( 35.6 ) |  | 7( 21.9 ) | 1( 9.1 ) | 1( 50 ) | 9( 20 ) |  |
|  | 4 | 0( 0 ) | 1( 5 ) | 1( 5.3 ) | 2( 4.4 ) |  | 11( 34.4 ) | 7( 63.6 ) | 1( 50 ) | 19( 42.2 ) |  |
|  | 5 | 1( 16.7 ) | 3( 15 ) | 2( 10.5 ) | 6( 13.3 ) |  | 14( 43.8 ) | 3( 27.3 ) | 0( 0 ) | 17( 37.8 ) |  |
| SE 1 | 1 | 2( 33.3 ) | 9( 45 ) | 11( 57.9 ) | 22( 48.9 ) | .571 |  |  |  |  | .559 |
|  | 2 | 4( 66.7 ) | 7( 35 ) | 4( 21.1 ) | 15( 33.3 ) |  |  |  |  |  |  |
|  | 3 | 0( 0 ) | 3( 15 ) | 3( 15.8 ) | 6( 13.3 ) |  | 14( 43.8 ) | 7( 63.6 ) | 1( 50 ) | 22( 48.9 ) |  |
|  | 4 | - | - | - | - |  | 9( 28.1 ) | 1( 9.1 ) | 1( 50 ) | 11( 24.4 ) |  |
|  | 5 | 0( 0 ) | 1( 5 ) | 1( 5.3 ) | 2( 4.4 ) |  | 9( 28.1 ) | 3( 27.3 ) | 0( 0 ) | 12( 26.7 ) |  |
| SE 2 | 1 | 4( 66.7 ) | 4( 20 ) | 12( 63.2 ) | 20( 44.4 ) | .084 | 3( 9.4 ) | 1( 9.1 ) | 0( 0 ) | 4( 8.9 ) | .354 |
|  | 2 | 1( 16.7 ) | 7( 35 ) | 4( 21.1 ) | 12( 26.7 ) |  |  |  |  |  |  |
|  | 3 | 0( 0 ) | 6( 30 ) | 3( 15.8 ) | 9( 20 ) |  | 5( 15.6 ) | 5( 45.5 ) | 1( 50 ) | 11( 24.4 ) |  |
|  | 4 | - | - | - | - |  | 8( 25 ) | 1( 9.1 ) | 1( 50 ) | 10( 22.2 ) |  |
|  | 5 | 1( 16.7 ) | 3( 15 ) | 0( 0 ) | 4( 8.9 ) |  | 16( 50 ) | 4( 36.4 ) | 0( 0 ) | 20( 44.4 ) |  |
|  |  |  |  |  |  |  |  |  |  |  |  |
| SE 3 | 1 | 2( 33.3 ) | 4( 20 ) | 9( 47.4 ) | 15( 33.3 ) | .341 | 8( 25 ) | 0( 0 ) | 0( 0 ) | 8( 17.8 ) | .149 |
|  | 2 | 3( 50 ) | 14( 70 ) | 7( 36.8 ) | 24( 53.3 ) |  |  |  |  |  |  |
|  | 3 | 1( 16.7 ) | 2( 10 ) | 3( 15.8 ) | 6( 13.3 ) |  | 5( 15.6 ) | 5( 45.5 ) | 1( 50 ) | 11( 24.4 ) |  |
|  | 4 | - | - | - | - |  | 8( 25 ) | 1( 9.1 ) | 1( 50 ) | 10( 22.2 ) |  |
|  | 5 | - | - | - | - |  | 11( 34.4 ) | 5( 45.5 ) | 0( 0 ) | 16( 35.6 ) |  |
| SE 4 | 1 | 2( 33.3 ) | 7( 35 ) | 9( 47.4 ) | 18( 40 ) | .705 | - | - | - | - | .537 |
|  | 2 | 3( 50 ) | 9( 45 ) | 4( 21.1 ) | 16( 35.6 ) |  | - | - | - | - |  |
|  | 3 | 1( 16.7 ) | 2( 10 ) | 3( 15.8 ) | 6( 13.3 ) |  | 14( 43.8 ) | 7( 63.6 ) | 1( 50 ) | 22( 48.9 ) |  |
|  | 4 | - | - | - | - |  | 10( 31.3 ) | 1( 9.1 ) | 1( 50 ) | 12( 26.7 ) |  |
|  | 5 | 0( 0 ) | 2( 10 ) | 3( 15.8 ) | 5( 11.1 ) |  | 8( 25 ) | 3( 27.3 ) | 0( 0 ) | 11( 24.4 ) |  |
| SE 5 | 1 | 2( 33.3 ) | 4( 20 ) | 11( 57.9 ) | 17( 37.8 ) | .907 | - | - | - | - | .456 |
|  | 2 | 3( 50 ) | 14( 70 ) | 5( 26.3 ) | 22( 48.9 ) |  | - | - | - | - |  |
|  | 3 | 1( 16.7 ) | 2( 10 ) | 3( 15.8 ) | 6( 13.3 ) |  | 14( 43.8 ) | 6( 54.5 ) | 1( 50 ) | 21( 46.7 ) |  |
|  | 4 | - | - | - | - |  | 8( 25 ) | 1( 9.1 ) | 1( 50 ) | 10( 22.2 ) |  |
|  | 5 | - | - | - | - |  | 10( 31.3 ) | 4( 36.4 ) | 0( 0 ) | 14( 31.1 ) |  |
| SE 6 | 1 | 1( 16.7 ) | 4( 20 ) | 10( 52.6 ) | 15( 33.3 ) | .251 | - | - | - | - | .221 |
|  | 2 | 3( 50 ) | 10( 50 ) | 5( 26.3 ) | 18( 40 ) |  | - | - | - | - |  |
|  | 3 | 2( 33.3 ) | 3( 15 ) | 3( 15.8 ) | 8( 17.8 ) |  | 5( 15.6 ) | 5( 45.5 ) | 1( 50 ) | 11( 24.4 ) |  |
|  | 4 | 0( 0 ) | 3( 15 ) | 1( 5.3 ) | 4( 8.9 ) |  | 19( 59.4 ) | 3( 27.3 ) | 1( 50 ) | 23( 51.1 ) |  |
|  | 5 | - | - | - | - |  | 8( 25 ) | 3( 27.3 ) | 0( 0 ) | 11( 24.4 ) |  |
| I1 | 1 | 1( 16.7 ) | 9( 45 ) | 3( 15.8 ) | 13( 28.9 ) | .200 |  |  |  |  | .744 |
|  | 2 | 4( 66.7 ) | 8( 40 ) | 7( 36.8 ) | 19( 42.2 ) |  |  |  |  |  |  |
|  | 3 | 1( 16.7 ) | 3( 15 ) | 8( 42.1 ) | 12( 26.7 ) |  | 1( 3.1 ) | 0( 0 ) | 0( 0 ) | 1( 2.2 ) |  |
|  | 4 | 0( 0 ) | 0( 0 ) | 1( 5.3 ) | 1( 2.2 ) |  | 16( 50 ) | 8( 72.7 ) | 1( 50 ) | 25( 55.6 ) |  |
|  | 5 | - | - | - | - |  | 15( 46.9 ) | 3( 27.3 ) | 1( 50 ) | 19( 42.2 ) |  |
| I2 | 1 | 1( 16.7 ) | 10( 50 ) | 3( 15.8 ) | 14( 31.1 ) | .313 | 1( 3.1 ) | 0( 0 ) | 0( 0 ) | 1( 2.2 ) | **.031*** |
|  | 2 | 4( 66.7 ) | 10( 50 ) | 10( 52.6 ) | 24( 53.3 ) |  | - | - | - | - |  |
|  | 3 | 1( 16.7 ) | 0( 0 ) | 6( 31.6 ) | 7( 15.6 ) |  | 7( 21.9 ) | 5( 45.5 ) | 2( 100 ) | 14( 31.1 ) |  |
|  | 4 | - | - | - | - |  | 10( 31.3 ) | 3( 27.3 ) | 0( 0 ) | 13( 28.9 ) |  |
|  | 5 | - | - | - | - |  | 14( 43.8 ) | 3( 27.3 ) | 0( 0 ) | 17( 37.8 ) |  |
| I3 | 1 | 1( 16.7 ) | 8( 40 ) | 3( 15.8 ) | 12( 26.7 ) | .269 |  |  |  |  | .680 |
|  | 2 | 4( 66.7 ) | 8( 40 ) | 8( 42.1 ) | 20( 44.4 ) |  |  |  |  |  |  |
|  | 3 | 1( 16.7 ) | 4( 20 ) | 8( 42.1 ) | 13( 28.9 ) |  | 8( 25 ) | 4( 36.4 ) | 1( 50 ) | 13( 28.9 ) |  |
|  | 4 | - | - | - | - |  | 10( 31.3 ) | 4( 36.4 ) | 1( 50 ) | 15( 33.3 ) |  |
|  | 5 | - | - | - | - |  | 14( 43.8 ) | 3( 27.3 ) | 0( 0 ) | 17( 37.8 ) |  |
| I4 | 1 | 1( 16.7 ) | 12( 60 ) | 5( 26.3 ) | 18( 40 ) | .056 |  |  |  |  | .682 |
|  | 2 | 4( 66.7 ) | 7( 35 ) | 8( 42.1 ) | 19( 42.2 ) |  |  |  |  |  |  |
|  | 3 | 1( 16.7 ) | 0( 0 ) | 6( 31.6 ) | 7( 15.6 ) |  |  |  |  |  |  |
|  | 4 | 0( 0 ) | 1( 5 ) | 0( 0 ) | 1( 2.2 ) |  | 9( 28.1 ) | 3( 27.3 ) | 0( 0 ) | 12( 26.7 ) |  |
|  | 5 | - | - | - | - |  | 23( 71.9 ) | 8( 72.7 ) | 2( 100 ) | 33( 73.3 ) |  |
| PB1 | 1 | 3( 50 ) | 9( 45 ) | 9( 47.4 ) | 21( 46.7 ) | .728 |  |  |  |  | .724 |
|  | 2 | 1( 16.7 ) | 8( 40 ) | 7( 36.8 ) | 16( 35.6 ) |  | 1( 3.1 ) | 0( 0 ) | 0( 0 ) | 1( 2.2 ) |  |
|  | 3 | 2( 33.3 ) | 2( 10 ) | 3( 15.8 ) | 7( 15.6 ) |  | 6( 18.8 ) | 2( 18.2 ) | 0( 0 ) | 8( 17.8 ) |  |
|  | 4 | 0( 0 ) | 1( 5 ) | 0( 0 ) | 1( 2.2 ) |  | 16( 50 ) | 4( 36.4 ) | 2( 100 ) | 22( 48.9 ) |  |
|  | 5 |  |  |  |  |  | 9( 28.1 ) | 5( 45.5 ) | 0( 0 ) | 14( 31.1 ) |  |
| PB2 | 1 | 3( 50 ) | 9( 45 ) | 7( 36.8 ) | 19( 42.2 ) | .680 |  |  |  |  | .820 |
|  | 2 | 1( 16.7 ) | 7( 35 ) | 5( 26.3 ) | 13( 28.9 ) |  | 1( 3.1 ) | 0( 0 ) | 0( 0 ) | 1( 2.2 ) |  |
|  | 3 | 2( 33.3 ) | 3( 15 ) | 7( 36.8 ) | 12( 26.7 ) |  | 6( 18.8 ) | 2( 18.2 ) | 0( 0 ) | 8( 17.8 ) |  |
|  | 4 | 0( 0 ) | 1( 5 ) | 0( 0 ) | 1( 2.2 ) |  | 18( 56.3 ) | 5( 45.5 ) | 2( 100 ) | 25( 55.6 ) |  |
|  | 5 |  |  |  |  |  | 7( 21.9 ) | 4( 36.4 ) | 0( 0 ) | 11( 24.4 ) |  |
| PB 3 | 1 | 3( 50 ) | 9( 45 ) | 7( 36.8 ) | 19( 42.2 ) | .758 |  |  |  |  | .820 |
|  | 2 | 1( 16.7 ) | 7( 35 ) | 5( 26.3 ) | 13( 28.9 ) |  | 1( 3.1 ) | 0( 0 ) | 0( 0 ) | 1( 2.2 ) |  |
|  | 3 | 2( 33.3 ) | 2( 10 ) | 5( 26.3 ) | 9( 20 ) |  | 6( 18.8 ) | 2( 18.2 ) | 0( 0 ) | 8( 17.8 ) |  |
|  | 4 | 0( 0 ) | 2( 10 ) | 2( 10.5 ) | 4( 8.9 ) |  | 18( 56.3 ) | 5( 45.5 ) | 2( 100 ) | 25( 55.6 ) |  |
|  | 5 |  |  |  |  |  | 7( 21.9 ) | 4( 36.4 ) | 0( 0 ) | 11( 24.4 ) |  |
| Total |  | 6( 100 ) | 20( 100 ) | 19( 100 ) | 45( 100 ) |  |  |  |  |  |  |

Supplementary table 1: Association of Behavioural constructs with Plaque index, * - Significant

|  |  | **PRE-INTERVENTION** | | | |  | **POST -INTERVENTION** | | | |  |
| --- | --- | --- | --- | --- | --- | --- | --- | --- | --- | --- | --- |
|  |  | **OHI-S INDEX** | | | |  | **OHI-S INDEX** | | | |  |
| Behavioural constructs | Likert  Scale score | **Good**  **n(%)** | **Fair**  **n(%)** | **Poor**  **n(%)** | **Total**  **n(%)** | **P value** | **Good**  **n(%)** | **Fair**  **n(%)** | **Poor**  **n(%)** | **Total**  **n(%)** | **P value** |
| OE1 | 1 | 1( 10 ) | 2( 8.3 ) | 2( 18.2 ) | 5( 11.1 ) | .618 |  |  |  |  | .104 |
|  | 2 | 6( 60 ) | 11( 45.8 ) | 4( 36.4 ) | 21( 46.7 ) |  | 0( 0 ) | 0( 0 ) | 1( 12.5 ) | 1( 2.2 ) |  |
|  | 3 | 3( 30 ) | 9( 37.5 ) | 4( 36.4 ) | 16( 35.6 ) |  | 7( 28 ) | 2( 16.7 ) | 0( 0 ) | 9( 20 ) |  |
|  | 4 | 0( 0 ) | 0( 0 ) | 1( 9.1 ) | 1( 2.2 ) |  | 9( 36 ) | 7( 58.3 ) | 6( 75 ) | 22( 48.9 ) |  |
|  | 5 | 0( 0 ) | 2( 8.3 ) | 0( 0 ) | 2( 4.4 ) |  | 9( 36 ) | 3( 25 ) | 1( 12.5 ) | 13( 28.9 ) |  |
| OE2 | 1 | 6( 60 ) | 6( 25 ) | 2( 18.2 ) | 14( 31.1 ) | .296 |  |  |  |  | .437 |
|  | 2 | 3( 30 ) | 7( 29.2 ) | 4( 36.4 ) | 14( 31.1 ) |  |  |  |  |  |  |
|  | 3 | 0( 0 ) | 9( 37.5 ) | 4( 36.4 ) | 13( 28.9 ) |  | 5( 20 ) | 3( 25 ) | 0( 0 ) | 8( 17.8 ) |  |
|  | 4 | 0( 0 ) | 1( 4.2 ) | 1( 9.1 ) | 2( 4.4 ) |  | 10( 40 ) | 6( 50 ) | 3( 37.5 ) | 19( 42.2 ) |  |
|  | 5 | 1( 10 ) | 1( 4.2 ) | 0( 0 ) | 2( 4.4 ) |  | 10( 40 ) | 3( 25 ) | 5( 62.5 ) | 18( 40 ) |  |
| OE3 | 1 | 1( 10 ) | 3( 12.5 ) | 1( 9.1 ) | 5( 11.1 ) | .954 |  |  |  |  | .774 |
|  | 2 | 3( 30 ) | 8( 33.3 ) | 4( 36.4 ) | 15( 33.3 ) |  |  |  |  |  |  |
|  | 3 | 4( 40 ) | 11( 45.8 ) | 4( 36.4 ) | 19( 42.2 ) |  | 5( 20 ) | 2( 16.7 ) | 1( 12.5 ) | 8( 17.8 ) |  |
|  | 4 | 1( 10 ) | 2( 8.3 ) | 1( 9.1 ) | 4( 8.9 ) |  | 10( 40 ) | 7( 58.3 ) | 3( 37.5 ) | 20( 44.4 ) |  |
|  | 5 | 1( 10 ) | 0( 0 ) | 1( 9.1 ) | 2( 4.4 ) |  | 10( 40 ) | 3( 25 ) | 4( 50 ) | 17( 37.8 ) |  |
| OE 4 | 1 | 4( 40 ) | 4( 16.7 ) | 1( 9.1 ) | 9( 20 ) | .255 |  |  |  |  | .476 |
|  | 2 | 3( 30 ) | 8( 33.3 ) | 4( 36.4 ) | 15( 33.3 ) |  |  |  |  |  |  |
|  | 3 | 1( 10 ) | 9( 37.5 ) | 4( 36.4 ) | 14( 31.1 ) |  | 6( 24 ) | 2( 16.7 ) | 2( 25 ) | 10( 22.2 ) |  |
|  | 4 | 0( 0 ) | 1( 4.2 ) | 2( 18.2 ) | 3( 6.7 ) |  | 8( 32 ) | 6( 50 ) | 5( 62.5 ) | 19( 42.2 ) |  |
|  | 5 | 2( 20 ) | 2( 8.3 ) | 0( 0 ) | 4( 8.9 ) |  | 11( 44 ) | 4( 33.3 ) | 1( 12.5 ) | 16( 35.6 ) |  |
| OE 5 | 1 | 1( 10 ) | 3( 12.5 ) | 1( 9.1 ) | 5( 11.1 ) | .213 |  |  |  |  | .973 |
|  | 2 | 4( 40 ) | 8( 33.3 ) | 4( 36.4 ) | 16( 35.6 ) |  |  |  |  |  |  |
|  | 3 | 1( 10 ) | 10( 41.7 ) | 5( 45.5 ) | 16( 35.6 ) |  | 5( 20 ) | 2( 16.7 ) | 2( 25 ) | 9( 20 ) |  |
|  | 4 | 0( 0 ) | 1( 4.2 ) | 1( 9.1 ) | 2( 4.4 ) |  | 10( 40 ) | 6( 50 ) | 3( 37.5 ) | 19( 42.2 ) |  |
|  | 5 | 4( 40 ) | 2( 8.3 ) | 0( 0 ) | 6( 13.3 ) |  | 10( 40 ) | 4( 33.3 ) | 3( 37.5 ) | 17( 37.8 ) |  |
| SE 1 | 1 | 5( 50 ) | 14( 58.3 ) | 3( 27.3 ) | 22( 48.9 ) | .096 |  |  |  |  | .339 |
|  | 2 | 5( 50 ) | 6( 25 ) | 4( 36.4 ) | 15( 33.3 ) |  |  |  |  |  |  |
|  | 3 | 0( 0 ) | 4( 16.7 ) | 2( 18.2 ) | 6( 13.3 ) |  | 12( 48 ) | 6( 50 ) | 4( 50 ) | 22( 48.9 ) |  |
|  | 4 |  |  |  |  |  | 4( 16 ) | 5( 41.7 ) | 2( 25 ) | 11( 24.4 ) |  |
|  | 5 | 0( 0 ) | 0( 0 ) | 2( 18.2 ) | 2( 4.4 ) |  | 9( 36 ) | 1( 8.3 ) | 2( 25 ) | 12( 26.7 ) |  |
| SE 2 | 1 | 4( 40 ) | 12( 50 ) | 4( 36.4 ) | 20( 44.4 ) | .499 | 3( 12 ) | 0( 0 ) | 1( 12.5 ) | 4( 8.9 ) | .688 |
|  | 2 | 5( 50 ) | 4( 16.7 ) | 3( 27.3 ) | 12( 26.7 ) |  |  |  |  |  |  |
|  | 3 | 1( 10 ) | 5( 20.8 ) | 3( 27.3 ) | 9( 20 ) |  | 5( 20 ) | 4( 33.3 ) | 2( 25 ) | 11( 24.4 ) |  |
|  | 4 |  |  |  |  |  | 4( 16 ) | 4( 33.3 ) | 2( 25 ) | 10( 22.2 ) |  |
|  | 5 | 0( 0 ) | 3( 12.5 ) | 1( 9.1 ) | 4( 8.9 ) |  | 13( 52 ) | 4( 33.3 ) | 3( 37.5 ) | 20( 44.4 ) |  |
|  |  |  |  |  |  |  |  |  |  |  |  |
| SE 3 | 1 | 3( 30 ) | 9( 37.5 ) | 3( 27.3 ) | 15( 33.3 ) | .416 | 6( 24 ) | 2( 16.7 ) | 0( 0 ) | 8( 17.8 ) | .480 |
|  | 2 | 7( 70 ) | 12( 50 ) | 5( 45.5 ) | 24( 53.3 ) |  |  |  |  |  |  |
|  | 3 | 0( 0 ) | 3( 12.5 ) | 3( 27.3 ) | 6( 13.3 ) |  | 5( 20 ) | 4( 33.3 ) | 2( 25 ) | 11( 24.4 ) |  |
|  | 4 |  |  |  |  |  | 4( 16 ) | 4( 33.3 ) | 2( 25 ) | 10( 22.2 ) |  |
|  | 5 |  |  |  |  |  | 10( 40 ) | 2( 16.7 ) | 4( 50 ) | 16( 35.6 ) |  |
| SE 4 | 1 | 3( 30 ) | 10( 41.7 ) | 5( 45.5 ) | 18( 40 ) | .412 |  |  |  |  | .649 |
|  | 2 | 5( 50 ) | 8( 33.3 ) | 3( 27.3 ) | 16( 35.6 ) |  |  |  |  |  |  |
|  | 3 | 0( 0 ) | 3( 12.5 ) | 3( 27.3 ) | 6( 13.3 ) |  | 11( 44 ) | 7( 58.3 ) | 4( 50 ) | 22( 48.9 ) |  |
|  | 4 |  |  |  |  |  | 6( 24 ) | 4( 33.3 ) | 2( 25 ) | 12( 26.7 ) |  |
|  | 5 | 2( 20 ) | 3( 12.5 ) | 0( 0 ) | 5( 11.1 ) |  | 8( 32 ) | 1( 8.3 ) | 2( 25 ) | 11( 24.4 ) |  |
| SE 5 | 1 | 4( 40 ) | 10( 41.7 ) | 3( 27.3 ) | 17( 37.8 ) | .448 |  |  |  |  | .782 |
|  | 2 | 6( 60 ) | 11( 45.8 ) | 5( 45.5 ) | 22( 48.9 ) |  |  |  |  |  |  |
|  | 3 | 0( 0 ) | 3( 12.5 ) | 3( 27.3 ) | 6( 13.3 ) |  | 13( 52 ) | 5( 41.7 ) | 3( 37.5 ) | 21( 46.7 ) |  |
|  | 4 |  |  |  |  |  | 4( 16 ) | 4( 33.3 ) | 2( 25 ) | 10( 22.2 ) |  |
|  | 5 |  |  |  |  |  | 8( 32 ) | 3( 25 ) | 3( 37.5 ) | 14( 31.1 ) |  |
| SE 6 | 1 | 3( 30 ) | 8( 33.3 ) | 4( 36.4 ) | 15( 33.3 ) | .944 |  |  |  |  | .623 |
|  | 2 | 5( 50 ) | 10( 41.7 ) | 3( 27.3 ) | 18( 40 ) |  |  |  |  |  |  |
|  | 3 | 1( 10 ) | 4( 16.7 ) | 3( 27.3 ) | 8( 17.8 ) |  | 5( 20 ) | 4( 33.3 ) | 2( 25 ) | 11( 24.4 ) |  |
|  | 4 | 1( 10 ) | 2( 8.3 ) | 1( 9.1 ) | 4( 8.9 ) |  | 12( 48 ) | 7( 58.3 ) | 4( 50 ) | 23( 51.1 ) |  |
|  | 5 |  |  |  |  |  | 8( 32 ) | 1( 8.3 ) | 2( 25 ) | 11( 24.4 ) |  |
| I1 | 1 | 2( 20 ) | 9( 37.5 ) | 2( 18.2 ) | 13( 28.9 ) | .604 |  |  |  |  | .542 |
|  | 2 | 6( 60 ) | 7( 29.2 ) | 6( 54.5 ) | 19( 42.2 ) |  |  |  |  |  |  |
|  | 3 | 2( 20 ) | 7( 29.2 ) | 3( 27.3 ) | 12( 26.7 ) |  | 0( 0 ) | 1( 8.3 ) | 0( 0 ) | 1( 2.2 ) |  |
|  | 4 | 0( 0 ) | 1( 4.2 ) | 0( 0 ) | 1( 2.2 ) |  | 15( 60 ) | 6( 50 ) | 4( 50 ) | 25( 55.6 ) |  |
|  | 5 |  |  |  |  |  | 10( 40 ) | 5( 41.7 ) | 4( 50 ) | 19( 42.2 ) |  |
| I2 | 1 | 2( 20 ) | 10( 41.7 ) | 2( 18.2 ) | 14( 31.1 ) | .605 | 0( 0 ) | 0( 0 ) | 1( 12.5 ) | 1( 2.2 ) | .313 |
|  | 2 | 6( 60 ) | 11( 45.8 ) | 7( 63.6 ) | 24( 53.3 ) |  |  |  |  |  |  |
|  | 3 | 2( 20 ) | 3( 12.5 ) | 2( 18.2 ) | 7( 15.6 ) |  | 8( 32 ) | 3( 25 ) | 3( 37.5 ) | 14( 31.1 ) |  |
|  | 4 |  |  |  |  |  | 8( 32 ) | 4( 33.3 ) | 1( 12.5 ) | 13( 28.9 ) |  |
|  | 5 |  |  |  |  |  | 9( 36 ) | 5( 41.7 ) | 3( 37.5 ) | 17( 37.8 ) |  |
| I3 | 1 | 2( 20 ) | 8( 33.3 ) | 2( 18.2 ) | 12( 26.7 ) | .707 |  |  |  |  | .513 |
|  | 2 | 6( 60 ) | 9( 37.5 ) | 5( 45.5 ) | 20( 44.4 ) |  |  |  |  |  |  |
|  | 3 | 2( 20 ) | 7( 29.2 ) | 4( 36.4 ) | 13( 28.9 ) |  | 7( 28 ) | 2( 16.7 ) | 4( 50 ) | 13( 28.9 ) |  |
|  | 4 |  |  |  |  |  | 9( 36 ) | 5( 41.7 ) | 1( 12.5 ) | 15( 33.3 ) |  |
|  | 5 |  |  |  |  |  | 9( 36 ) | 5( 41.7 ) | 3( 37.5 ) | 17( 37.8 ) |  |
| I4 | 1 | 2( 20 ) | 13( 54.2 ) | 3( 27.3 ) | 18( 40 ) | .295 |  |  |  |  | .572 |
|  | 2 | 6( 60 ) | 8( 33.3 ) | 5( 45.5 ) | 19( 42.2 ) |  |  |  |  |  |  |
|  | 3 | 2( 20 ) | 3( 12.5 ) | 2( 18.2 ) | 7( 15.6 ) |  |  |  |  |  |  |
|  | 4 | 0( 0 ) | 0( 0 ) | 1( 9.1 ) | 1( 2.2 ) |  | 7( 28 ) | 4( 33.3 ) | 1( 12.5 ) | 12( 26.7 ) |  |
|  | 5 |  |  |  |  |  | 18( 72 ) | 8( 66.7 ) | 7( 87.5 ) | 33( 73.3 ) |  |
| PB1 | 1 | 6( 60 ) | 11( 45.8 ) | 4( 36.4 ) | 21( 46.7 ) | .829 |  |  |  |  | .945 |
|  | 2 | 2( 20 ) | 9( 37.5 ) | 5( 45.5 ) | 16( 35.6 ) |  | 1( 4 ) | 0( 0 ) | 0( 0 ) | 1( 2.2 ) |  |
|  | 3 | 2( 20 ) | 3( 12.5 ) | 2( 18.2 ) | 7( 15.6 ) |  | 4( 16 ) | 3( 25 ) | 1( 12.5 ) | 8( 17.8 ) |  |
|  | 4 | 0( 0 ) | 1( 4.2 ) | 0( 0 ) | 1( 2.2 ) |  | 13( 52 ) | 5( 41.7 ) | 4( 50 ) | 22( 48.9 ) |  |
|  | 5 |  |  |  |  |  | 7( 28 ) | 4( 33.3 ) | 3( 37.5 ) | 14( 31.1 ) |  |
| PB2 | 1 | 4( 40 ) | 11( 45.8 ) | 4( 36.4 ) | 19( 42.2 ) | .469 |  |  |  |  | .870 |
|  | 2 | 1( 10 ) | 8( 33.3 ) | 4( 36.4 ) | 13( 28.9 ) |  | 1( 4 ) | 0( 0 ) | 0( 0 ) | 1( 2.2 ) |  |
|  | 3 | 5( 50 ) | 4( 16.7 ) | 3( 27.3 ) | 12( 26.7 ) |  | 4( 16 ) | 3( 25 ) | 1( 12.5 ) | 8( 17.8 ) |  |
|  | 4 | 0( 0 ) | 1( 4.2 ) | 0( 0 ) | 1( 2.2 ) |  | 15( 60 ) | 5( 41.7 ) | 5( 62.5 ) | 25( 55.6 ) |  |
|  | 5 |  |  |  |  |  | 5( 20 ) | 4( 33.3 ) | 2( 25 ) | 11( 24.4 ) |  |
| PB 3 | 1 | 4( 40 ) | 11( 45.8 ) | 4( 36.4 ) | 19( 42.2 ) | .466 |  |  |  |  | .870 |
|  | 2 | 1( 10 ) | 8( 33.3 ) | 4( 36.4 ) | 13( 28.9 ) |  | 1( 4 ) | 0( 0 ) | 0( 0 ) | 1( 2.2 ) |  |
|  | 3 | 3( 30 ) | 3( 12.5 ) | 3( 27.3 ) | 9( 20 ) |  | 4( 16 ) | 3( 25 ) | 1( 12.5 ) | 8( 17.8 ) |  |
|  | 4 | 2( 20 ) | 2( 8.3 ) | 0( 0 ) | 4( 8.9 ) |  | 15( 60 ) | 5( 41.7 ) | 5( 62.5 ) | 25( 55.6 ) |  |
|  | 5 |  |  |  |  |  | 5( 20 ) | 4( 33.3 ) | 2( 25 ) | 11( 24.4 ) |  |
| Total |  | 6( 100 ) | 20( 100 ) | 19( 100 ) | 45( 100 ) |  | 25( 100 ) | 12( 100 ) | 8( 100 ) | 45( 100 ) |  |

Supplementary table 2: Association of Behavioural constructs with OHI-S
